# Posaconazole inhibits the stemness of cancer stem-like cells by inducing autophagy and suppressing the Wnt/β-catenin/survivin signaling pathway in glioblastoma

**DOI:** 10.3389/fphar.2022.905082

**Published:** 2022-08-11

**Authors:** Hua Wang, Yinfeng Tan, Hao Jia, Danqi Liu, Rangru Liu

**Affiliations:** ^1^ Key Laboratory of Tropical Translational Medicine of Ministry of Education, NHC Key Laboratory of Control of Tropical Diseases, School of Pharmacy, Department of Spine Surgery of The First Affiliated Hospital, Hainan Medical University, Haikou, China; ^2^ Hainan Provincial Key Laboratory for Research and Development of Tropical Herbs, Haikou Key Laboratory of Li Nationality Medicine, School of Pharmacy, Hainan Medical University, Haikou, China; ^3^ Department of Pharmacy, Xiangya Hospital, Central South University, Changsha, China

**Keywords:** posaconazole, cancer stem-like cells, autophagy, survivin, Wnt/β-catenin signaling pathway

## Abstract

Posaconazole (POS) has been reported to present potential antitumor activity for glioblastoma (GBM). However, its molecular mechanisms remain unclear. In this study, we found that POS has potent cytotoxicity and inhibits cell viability and proliferation in GBM. In addition, we adopted a sphere formation assay to detect the self-renewal capacity, performed western blotting to measure cancer stem-like cells (CSCs) marker proteins (CD133, SOX2, Nanog and Oct4) and applied flow cytometry to monitor the subpopulation of CD144^+^/CD33^+^ cells, and the results all demonstrated that POS can remarkably weaken CSCs stemness. Furthermore, western blotting, immunoflurescence, transmission electron microscopy and acridine orange staining were performed to detect autophagy-related proteins (LC3, SQSTM1, Beclin 1 and Atg5), count the numbers of endogenous LC3 puncta, visually observe the ultrastructural morphology of autophagosomes and judge the formation of acidic vesicular organelles, respectively, and the results validated that POS promotes autophagy induction. Importantly, the suppressive effect of POS on CSCs stemness was partially relieved when autophagy was blocked by the autophagy inhibitor chloroquine (CQ) or Atg5 shRNA. Bioinformatic techniques, including weighted gene coexpression network analysis (WGCNA), gene set difference analysis (GSVA) and KEGG pathway analysis, combined with experimental validations showed that survivin, which is implicated in both autophagy and the stem cell index, is one of the target proteins of POS and that POS weakens CSCs stemness via suppressing the Wnt/β-catenin signaling pathway in GBM. Besides, POS-induced autophagy and the Wnt/β-catenin signaling pathway are negative regulators for each other. Finally, the antitumor activity of POS was confirmed in GBM xenograft models *in vivo*. Consistent with the *in vitro* conclusions, POS upregulated the expression of LC3 and decreased the expression of CD133, survivin and β-catenin, as shown by the immunohistochemistry analysis. In summary, this work provides an experimental foundation for exploiting POS as a CSCs-targeting antitumor drug for GBM treatment.

## Introduction

Glioblastoma (GBM, classified as grade Ⅳ according to the World Health Organization [WHO]) is the most common and lethal primary malignant intracranial tumor in adults (Stupp et al., 2009). The current standard treatment for GBM involves surgical resection followed by radiotherapy plus chemotherapy (Stupp et al., 2005; Anton et al., 2012). Despite this multiple-combination of therapeutic methods, recurrence is inevitable, and the overall prognosis for GBM remains extremely poor, with a median survival of 14.6 months and an average 5-year survival rate of 5% (Stupp et al., 2017). Therefore, it is urgent to develop more effective therapeutic strategies to treat GBM in the clinic.

One major problem in treating GBM is the occurrence of cancer stem-like cells (CSCs), which may represent a specific subpopulation of GBM cells. CSCs have the capacity to self-renew, express stem cell markers, form tumor spheres, differentiate into multiple lineages and phenocopy the original tumor *in vivo* (Eyler et al., 2011; Flavahan et al., 2013). Increasing evidence has shown that CSCs are closely related to tumor recurrence and resistance to conventional therapy (Reya et al., 2001; Chen et al., 2012; Wang et al., 2018). Therefore, it can be assumed that powerful control of GBM can be achieved by specifically targeting CSCs.

Posaconazole (POS) is a second-generation broad-spectrum antifungal triazole drug, that is, mainly used for the treatment of mycoemia and respiratory, digestive and urinary tract mycosis, peritonitis, and meningitis caused by *candida* and *crytococcus* fungi (Schein et al., 2011). Antifungal agents especially azoles have recently displayed potential anti-GBM activities (Poser et al., 2019; Lyne and Yamini, 2021). A drug-screening study found that POS could markedly weaken GBM cell viability and inhibit cell proliferation due to its ability to inhibit hexokinase II. Moreover, POS successfully crossed the blood-brain barrier, which suggested that POS could arrive at the brain after oral administration (Agnihotri et al., 2019). Thus, POS has huge potential and bright prospects for clinical translation in GBM treatment. At present, there is an ongoing early Phase I trial which investigates the therapeutic efficacy of posaconazole and ketoconazole in recurrent High grade gliomas (HGG, WHO grade III and IV) patients (University Health Network, 2018). However, current understanding of the antitumor mechanisms for POS against CSCs in GBM remains limited.

Autophagy is a biological process in which aging or damaged organelles and biomolecules are degraded and reused in cells. The autophagy process can be divided into several key stages, including the formation of autophagosomes, the inclusion of cargo, the fusion of autophagosomes with lysosomes to form autolysosomes, and the degradation of cellular cargo (Marino et al., 2014). An increasing number of studies have shown that autophagy is closely implicated in CSCs functions and could represent a “double-edged sword” in the survival or maintenance of CSCs (Bellodi et al., 2009; Kumar et al., 2013; Zhang et al., 2016; Pagotto et al., 2017; Sharif et al., 2017; Zhang et al., 2017). Therefore, clarifying the specific relationship between autophagy and CSCs and further illuminating the effect of autophagy in regulating CSCs are important research topics for targeting CSCs in cancer treatment. It is unclear until now whether POS can induce autophagy in GBM. If it does, the role of POS-induced autophagy in CSCs stemness deserves further exploration.

In this study, we found that POS effectively inhibits cell viability, cell proliferation and CSCs stemness in GBM. More notably, POS remarkably induces autophagy and the autophagy partially neutralizes the inhibitory effect of POS on CSCs stemness in GBM. Our study also discovered that POS weakens CSCs stemness by targeting survivin and suppressing the Wnt/β-catenin signaling pathway. Furthermore, POS-induced autophagy intersects with the Wnt/β-catenin pathway. Finally, the antitumor activity of POS was confirmed in GBM xenograft models *in vivo*. Our work offers an experimental foundation for exploiting POS as a CSCs-targeting antitumor drug for GBM treatment.

## Materials and methods

### Cell lines and cell treatment

Two GBM cell lines, U251 and U87-MG, were obtained from the Shanghai Cell Bank of the Chinese Academy of Sciences. The two cell lines were cultured in Dulbecco’s modified Eagle’s medium (DMEM) supplemented with fetal bovine serum (10%, Gibco) and penicillin-streptomycin (10U/ml, HyClone) in an incubator (5% CO_2_, 37°C). Cells were treated with the prescribed concentrations of POS before cell harvesting and subsequent cell detection.

### Reagents and antibodies

All reagents and antibodies adopted in this study were purchased from Chinese suppliers (in parentheses): POS (MCE, HY-17373), chloroquine (Sigma, C6628), acridine orange (Sigma, 158550), dimethyl sulfoxide (DMSO, Sigma, 67–68-5), Cell Counting Kit-8 (CCK-8, Dojindo, CK04), Cell Cycle Detection Kit (KeyGEN, KGA512), BrdU Cell Proliferation Assay Kit (Abcam, ab287841), recombinant human EGF protein (Abcam, ab9697), recombinant human FGF protein (Abcam, ab9596), B-27 (Gibco, 17504–044), N-2 (Gibco, 17502–048), CD133 (Abcam, ab19898), SOX2 (Abcam, ab97959), Nanog (Abcam, ab21624), Oct4 (Abcam, ab19857), Beclin1 (Abcam, ab62557), Atg5 (Abcam, ab108327), survivin (Abcam, ab76424), Wnt1 (Abcam, ab15251), β-catenin (Abcam, ab32572), c-Myc (Abcam, ab32072), CD44-FITC (Abcam, ab27285), CD133-APC (Abcam, ab253259), SQSTM1 (MBL, PM045) and LC3 (MBL, PM036).

### Inhibition of ATG5 and overexpression of survivin and β-catenin

The lentiviral vector, shRNA sequences targeting human Atg5, human survivin and β-catenin overexpression plasmids were purchased from GenePharma (Shanghai, China). Lentiviral infection was performed according to the directions provided by GenePharma, and the inhibition efficacy of shAtg5 and the overexpression efficacy of survivin and β-catenin were confirmed by western blot analysis (Figure 4C and Supplementary Figures 2SA, 2SB).

### Cell viability detection

About 3,000 cells were seeded into 96-well plates overnight and then treated with POS at the prescribed concentrations. After treatment for 24 h, the cells was added with cell counting kit-8 (CCK-8, 10μL/well), incubated for 2 h at 37°C and subsequently detected O.D values at 450 nm regarding the culture medium without cells as the blank control. Cell viability was indicated as the percentage of absorbance in the cells that experienced POS treatment versus those that experienced DMSO treatment, which was considered the control group.

### Bromodeoxyuridine cell proliferation assay

A bromodeoxyuridine (BrdU) Cell Proliferation ELISA Kit was adopted to detect cell proliferation. About 2×10^4^ cells were seeded into 96-well plates in triplicate and incubated overnight. Then, the cells were treated with POS for 24 h with DMSO groups as the controls. BrdU (20 μL/well) was added and incubated for 12 h. Then fixing solution, primary detector antibody, HRP conjugate antibody, the TMB solution and the stop solution were added in sequence. O.D values at 450 nm were recorded by a microplate reader. Cell proliferation is presented as the percentage of BrdU incorporation in the cells that endured POS treatment versus those that endured DMSO treatment.

### Clone formation

About 500 cells were seeded in 6-well plates and treated with POS at the indicated concentrations for approximately 2 weeks with DMSO groups as the controls. The formed cell clones were stained with crystal violet and counted by a microscope. Clones were defined when the number of clustered cells was over 50.

### Cell cycle detection

A Cell Cycle Detection Kit was used to detect the cell cycle distribution. Appropriate number of cells were seeded into 6-well plates and then treated with POS at the prescribed concentrations for 24 h. The cells were fixed with 70% (v/v) cold ethyl alcohol at 4°C for overnight and stained with 500 μL RNase A/PI staining solution (v/v, 1:9) in the dark at room temperature for 30 min. The cell DNA content was tested by flow cytometry (Sysmex, North Rhine-Westphalia, Germany) and analyzed with FCS Express software version 6.

### Western blot analysis

Proteins were extracted using RIPA buffer and quantified by a BCA kit. Protein lysates were separated using 10% SDS-PAGE and transferred to PVDF membranes. After blocking, the PVDF membranes were incubated with the primary antibodies and the secondary antibodies. The target proteins were imaged with enhanced chemiluminescence reagents (Merck Millipore, Billerica, MA).

### Flow cytometry analysis

Appropriate number of cells were seeded in 6-well plates and treated with POS at 5, 10 and 20 μM for 24 h with DMSO groups as the controls. Cells were incubated with 10 μL CD44-FITC and CD133-APC in the dark for 30 min. After washing with PBS three times, the cells were detected by flow cytometry (Sysmex) and analyzed with FCS Express version software 6.

### Sphere formation assay

A total of 2000 cells were seeded and treated as indicated in 24-well ultralow cluster plates (Corning) for approximately 14 days. Spheres were cultured in DMEM/F12 serum-free medium (HyClone) supplemented with EGF, FGF, B-27 and N-2. Finally, the formed spheres were observed and photographed under a microscope.

### Acridine orange staining

Cells were seeded in 6-well plates overnight and treated with DMSO or POS as indicated. Then, the treated cells were digested, harvested, and stained with acridine orange (AO) solution (1 μg/ml). After washing with PBS, the cells were detected by flow cytometry (Sysmex). The results were handled by FCS Express software version 6.

### Immunofluorescence

Cells were successively treated with 4% paraformaldehyde, 0.5%Triton X-100 and 5% bovine serum albumin for cell fixation, permeabilization and blocking, respectively. After washing with PBS, the cells were incubated with LC3 antibodies and goat anti-rabbit IgG H&L, the nuclei were stained with DAPI, and the cells were photographed by laser scanning confocal microscopy (Olympus FV1000, Tokyo, Japan).

### Bioinformatics analysis

The RNA-sequencing gene expression profiles of 156 primary GBM tumor tissues and 5 normal tissues were downloaded from The Cancer Genome Atlas (TCGA) and subjected to differential expression analysis by using the “edge R” package of R language according to the following screening criteria: |logFC| > 1 and FDR < 0.05. Then, 6,648 differentially expressedgenes were screened out. We selected the top 5,000 genes with the largest median absolute deviation (MAD) for WGCNA by adopting the “WGCNA” package of R language based on the screening criteria: cor. gene MM > 0.8 and cor. gene GS > 0.5, and we then explored the associated modules with the stem cell characteristic index, namely, mRNAsi and EREG-mRNAsi (Malta et al., 2018). Gene set enrichment analysis (GSEA) can judge whether statistically significant differential expression occurs between high and low gene expression groups, and the phenotypes were labeled as survivn-high and survivn-low. The Kyoto Encyclopedia of Genes and Genomes (KEGG) website (https://www.kegg.jp/) was employed to determine the related pathway map based on the keyword survivin.

### Animal study

U87-MG cells were subcutaneously injected in immunodeficient nude mice at 6–8 weeks of age to establish glioblastoma xenograft models. Mice were randomly divided into 2 groups (5 mice in each group) and injected through the tail vein with DMSO and POS (50 mg/kg) once every 3 days until the 18th day. The tumor volumes were measured every 3 days by using a handheld imaging device (TM900, Peira, Belgium). The tumor tissues were harvested for immunohistochemistry.

### Immunohistochemistry

Immunohistochemistry was performed on 4% paraformaldehyde-fixed, paraffin-embedded sections. Briefly, following conventional dewaxing and hydration, the sections were immersed in sodium citrate (0.01 M, pH 6.0) at 60°C overnight for antigen retrieval. After covered with 3% hydrogen peroxide for 10 min in the dark, the sections were permeabilized and blocked in 1% goat serum supplemented with 0.2% Triton X-100 (Beyotime, Shanghai, China) at room temperature for 30 min. Subsequently, anti-LC3, CD133, survivin, and β-catenin were used as the primary antibodies. Then, the slides were stained with HRP (horse-radish peroxidase)-conjugated goat anti-rabbit IgG H&L (Proteintech Group, IL, United States) at 37°C for 60 min followed by visualization with 3, 3-diaminobenzidine (DAB) (ZSGB-Bio, Beijing, China). The microscopic observation was performed using an Olympus IX50 (Olympus, Tokyo, Japan).

### Statistical analysis

Statistical analyses were conducted with SPSS Statistics software version 20. The values were compared by one-way ANOVA or independent-samples Student’s *t* test. Statistical significance was determined at *p < 0.05, **p < 0.01, or ***p < 0.001. Values are presented as the mean ± SEM. Error bars indicate the SEM unless otherwise noted.

## Results

### Posaconazole has potent cytotoxicity and retards the cell cycle in glioblastoma cells

**FIGURE 1:**
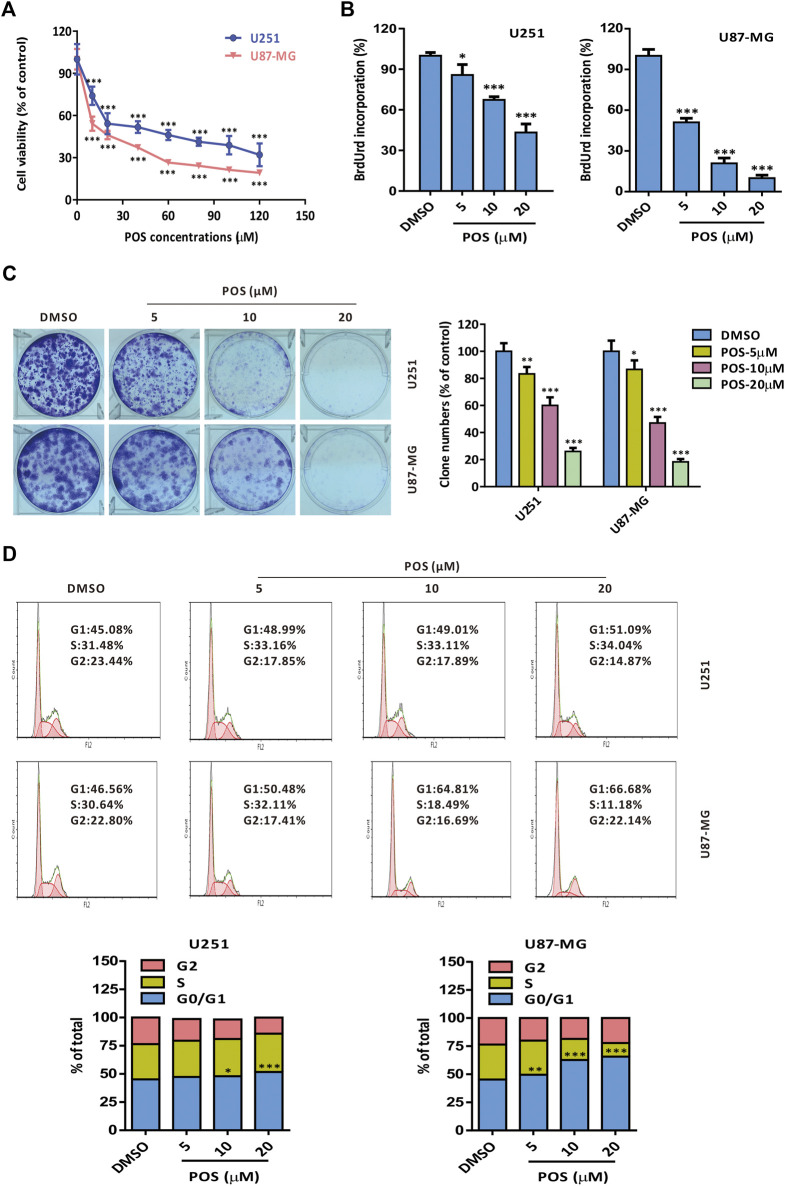
POS shows potent cytotoxicity and retards the cell cycle in GBM cells. **(A)** Cell viability was detected by CCK-8 assay after the cells were treated as indicated for 24 h. **(B)** Cell proliferation was measured by BrdU labeling assay after the cells were handled as indicated for 24 h. **(C)** Long-term cell proliferation was observed by colony formation assay after the cells were treated as indicated for 2 weeks. **(D)** Cell cycle was monitored by a Cell Cycle Detection Kit after the cells were handled as indicated for 24 h.

To investigate the antitumor effect of POS on GBM, two GBM cell lines, U251 and U87-MG, were incubated with POS at the prescribed concentrations for 24 h and then CCK-8 assay was used to detect cell viability. The results showed that POS dose-dependently attenuated the viability of GBM cells (Figure 1A). A similar conclusion was obtained from BrdU labeling assay (Figure 1B) and colony formation assay (Figure 1C). Furthermore, to examine whether POS could affect cell cycle progression in GBM cells, a Cell Cycle Detection Kit was used to observe the cell cycle distribution. POS significantly increased the cell population at the G_0_/G_1_ phase accompanied by reduced cell numbers at the S and G_2_/M phases in a dose-dependent manner after treatment for 24 h (Figure 1D). In short, the above results showed that POS inhibits cell proliferation and retards the cell cycle in GBM cells.

### Posaconazole attenuates cancer stem-like cells stemness in glioblastoma cells

**FIGURE 2:**
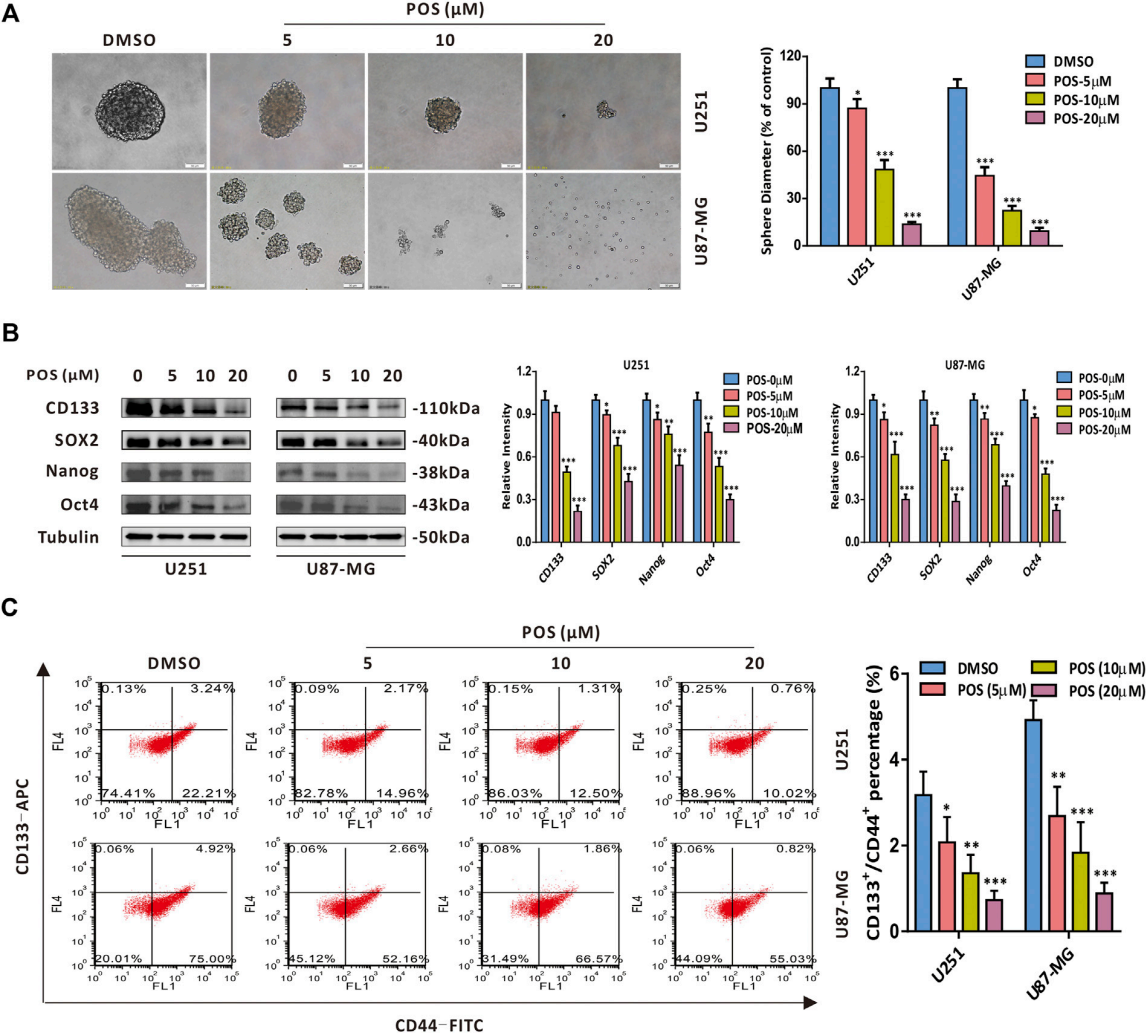
POS attenuates CSCs stemness in GBM cells. **(A)** Self-renewal capacity was detected by a sphere formation assay after the cells were treated as indicated for 2 weeks. Scale bar, 50 μM. **(B)** Expression levels of the CSCs marker proteins CD133, SOX2, Nanog and Oct4 were measured by western blotting after the cells were treated as indicated for 24 h. **(C)** CD44^+^/CD133^+^ subpopulations were monitored by flow cytometry after the cells were handled as in **(B)**.

To explore whether POS plays a role in CSCs stemness in GBM cells, we first performed a sphere formation assay to detect the self-renewal capacity. The results showed that the size and number of spheres were dose-dependently decreased in POS-treated U251 and U87-MG cells compared with the control cells (Figure 2A). Moreover, CSCs marker proteins, including CD133, SOX2, Nanog and Oct4, were downregulated in a dose-dependent manner in POS-treated GBM cells (Figure 2B). Given that subpopulations of CD44^+^/CD133^+^ surface marker cells exhibit significant stem cell characteristics and self-renewal capacity (Brown et al., 2015; Brown et al., 2017), we further investigated whether POS could influence CD44^+^/CD133^+^ subpopulations in GBM cells. Flow cytometry showed that POS dose-dependently decreased CD44^+^/CD133^+^ subpopulations after POS treatment (Figure 2C). Taken together, these data indicated that POS weakens CSCs stemness in GBM cells.

### Posaconazole induces autophagy in glioblastoma cells

**FIGURE 3:**
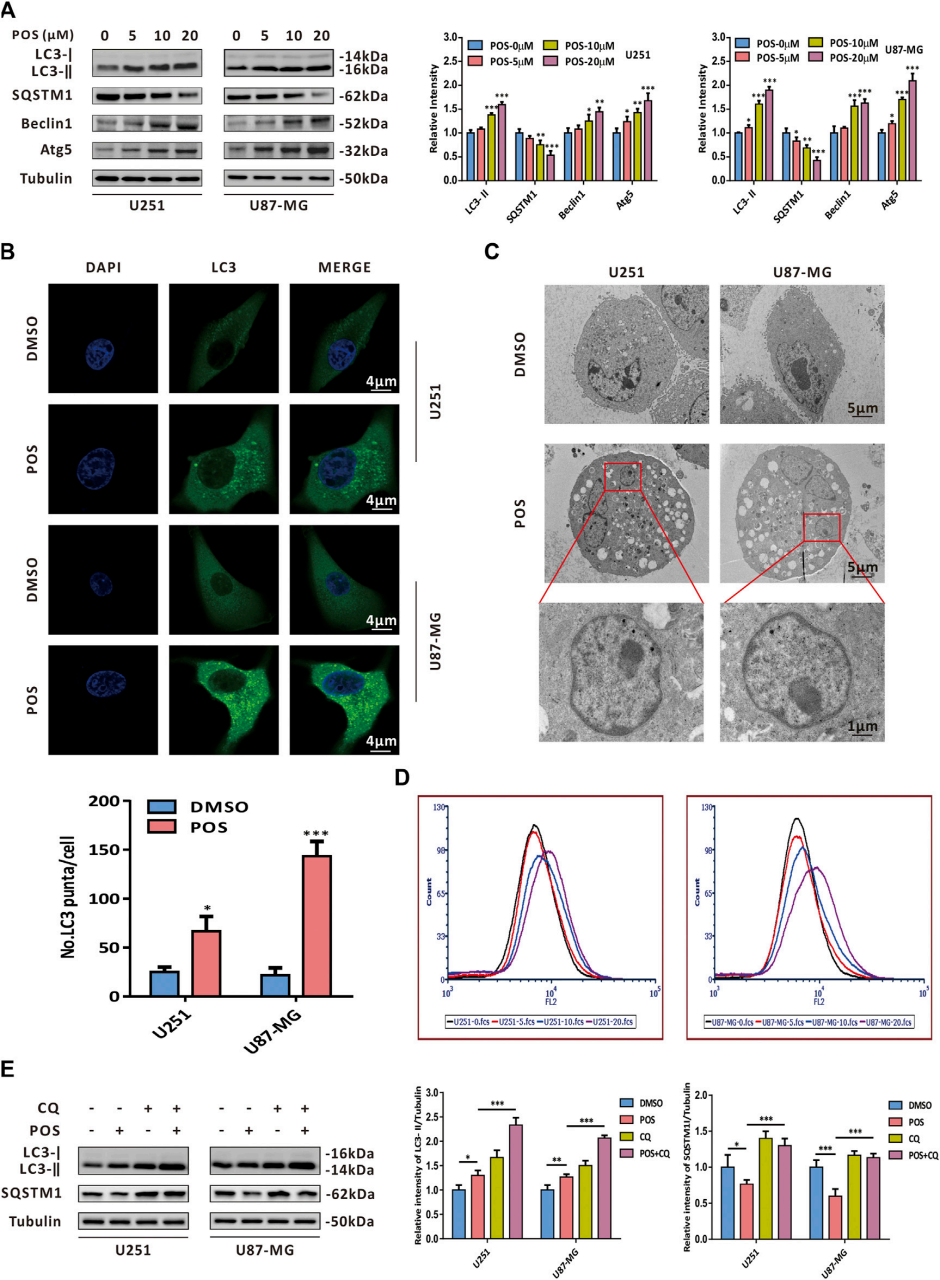
POS provokes autophagy in GBM cells. **(A)** Expression levels of the autophagy marker proteins LC3, SQSTM1, Beclin1 and Atg5 were detected by western blotting after the cells were treated as indicated for 24 h. **(B)** Endogenous LC3 puncta were observed by immunofluorescence after the cells were handled as in **(A)**. Scale bar, 4 μM. **(C)** Autophagosomes/autolysosomes were observed by transmission electron microscopy after the cells were handled as in **(A)**. Scale bar: the upper and middle, 5 μM; the lower, 1 μM. **(D)** Formation of AVOs was tested by AO staining after the cells were treated as in **(A)**. **(E)** Expression levels of LC3 and SQSTM1 were detected by western blotting after the cells were treated with or without POS in the presence or absence of CQ.

With a growing number of studies confirming the role of autophagy in chemotherapeutics (Bellodi et al., 2009; Kumar et al., 2013; Zhang et al., 2016; Pagotto et al., 2017; Sharif et al., 2017; Zhang et al., 2017), we next studied whether POS could induce autophagy in GBM cells. To this end, western blotting was conducted to detect the expression levels of autophagy-related proteins. The western blot analysis showed that POS dose-dependently increased LC3-II, Beclin1 and Atg5 expression levels but had the opposite effect on the SQSTM1 protein in both U251 and U87-MG cells (Figure 3A). In addition, POS obviously enhanced the numbers of endogenous LC3 puncta when compared with the control groups (Figure 3B). Moreover, transmission electron microscopy (TEM) showed that compared with the controls, POS-treated cells possessed a double-membraned vacuolar structure, which is the ultrastructural morphological characteristic of autophagosomes (Figure 3C). Acridine orange (AO) staining was also applied to detect another classical characteristic of autophagy, acidic vesicular organelles (AVOs). AO staining showed that POS promoted the formation of AVOs compared with the controls (Figure 3D).

To further investigate whether POS could facilitate autophagic flux, GBM cells were treated with POS in the presence or absence of the autophagy inhibitor chloroquine (CQ). Then, we conducted western blotting assay to detect the expression levels of LC3 and SQSTM1. The increased expression levels of LC3-Ⅱ and SQSTM1 in POS plus CQ treated cells compared with POS-treated cells alone validated that POS could promote intact autophagic flux in GBM cells (Figure 3E).

All of the above data clearly verified that POS induces autophagy in GBM cells.

### Posaconazole impairs cancer stem-like cells stemness through inducing autophagy in glioblastoma cells

**FIGURE 4:**
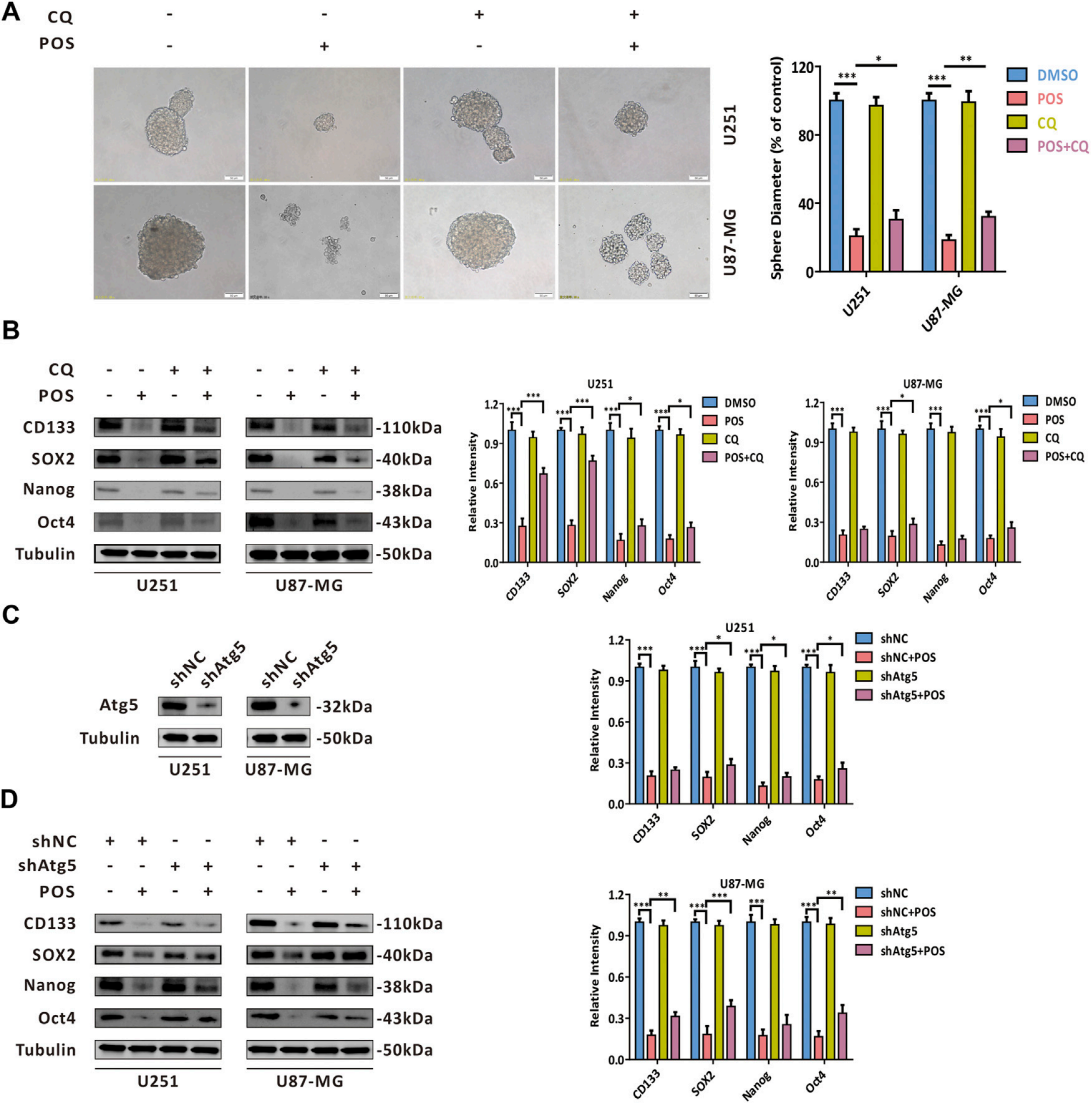
POS impairs CSCs stemness via inducing autophagy in GBM cells. **(A)** Self-renewal capacity was detected by a sphere formation assay after the cells were treated with or without POS in the presence or absence of CQ for 2 weeks. Scale bar, 50 μM. **(B)** Expression levels of the CSCs marker proteins CD133, SOX2, Nanog and Oct4 were measured by western blotting after the cells were treated with or without POS in the presence or absence of CQ for 24 h. **(C)** Efficacy of Atg5 shRNA was validated by western blotting. **(D)** Expression levels of the CSCs marker proteins CD133, SOX2, Nanog and Oct4 were examined by western blotting after the Atg5 shRNA or shNC cells were treated with or without POS for 24 h.

Given that autophagy participates in multiple physiological and pathological processes, we sought to determine whether POS-induced autophagy could play a role in weakening CSCs stemness in GBM cells. As shown in Figure 4A, after the autophagy inhibitor CQ was adopted to block POS-induced autophagy, the capacity to form spheres was partially restored compared with the POS treatment alone. Consistently, POS plus CQ partially counteracted the downregulatory effect of POS on the expression levels of CSCs marker proteins, including CD133, SOX2, Nanog and Oct4 (Figure 4B). Atg5 shRNA was also adopted to block the POS-induced autophagy. The inhibition efficacy shAtg5 was confirmed by western blotting (Figure 4C). Similar conclusions were obtained by using Atg5 shRNA rather than by an autophagy inhibitor (Figure 4D). Besides, clone formation results indicated that POS plus CQ remarkably promoted clonogenic survival compared with POS alone (Supplementary Figure S1).

In short, the above results indicated that POS weakens CSCs stemness partially by inducing autophagy in GBM cells.

### Posaconazole targets survivin to attenuate cancer stem-like cells stemness in glioblastoma cells

**FIGURE 5:**
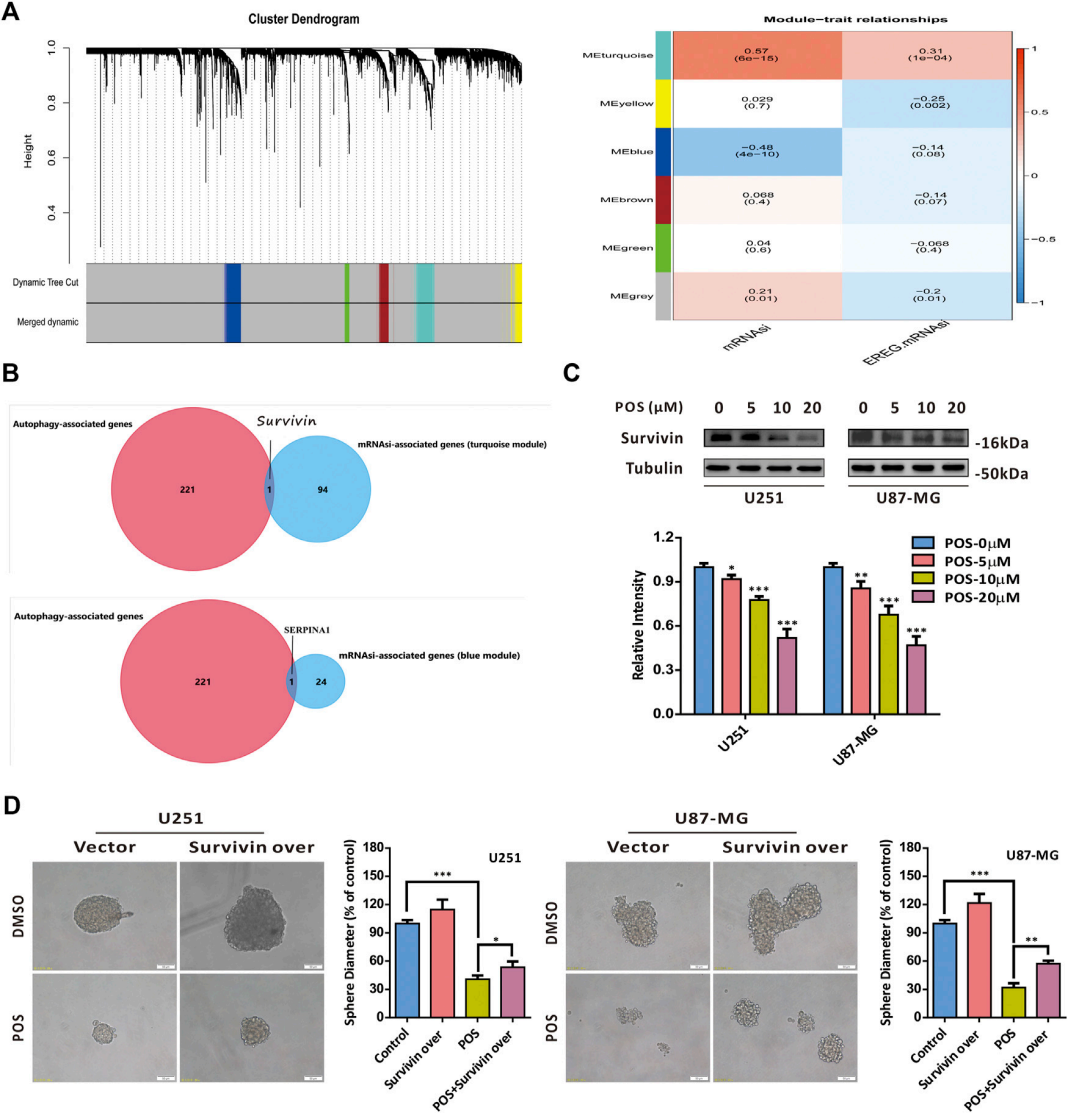
POS targets survivin to weaken CSCs stemness in GBM cells. **(A)** WGCNA was applied to analyze gene expression profiles from the TCGA database to find coexpression modules related to the stem cell index (mRNAsi and EREG-mRNAsi) in GBM patients compared to healthy controls. **(B)** Results of **(A)** were intersected with the autophagy-related genes provided by the human autophagy database HADb. **(C)** Survivin expression was detected by western blotting after cells were treated as indicated for 24 h. **(D)** Self-renewal capacity was detected by a sphere formation assay after the survivin-overexpressed or vector control cells were treated with POS or DMSO for 2 weeks. Scale bar, 50 μM.

To screen out the target proteins of POS in GBM cells, we first applied weighted gene coexpression network analysis (WGCNA) to GBM gene expression data from The Cancer Genome Atlas (TCGA) database to explore key coexpression modules related to stem cell indexes (mRNAsi and EREG-mRNAsi) in GBM patients compared to healthy controls. As a result, we found that two coexpression modules, turquoise and blue modules, were significantly associated with the stem cell index (Figure 5A and Supplementary Table S1). Given that POS could induce autophagy in GBM cells, to narrow the screening scope, we further intersected with the autophagy-related genes provided by the human autophagy database HADb (https://www.autophagy.lu/). Therefore, survivin (also named BIRC5) and SERPINA1 were selected as candidate target proteins for POS in GBM cells (Figure 5B). Recent studies have reported that survivin is closely related to CSCs in cancers (Kanwar et al., 2015; Wang et al., 2015). Therefore, we chose survivin as a potential target protein for POS. As a validation, POS downregulated survivin expression in a dose-dependent manner in GBM cells (Figure 5C). Notably, sphere formation experiments indicated that survivin overexpression partially offset the inhibitory effect of POS on the capacity to form spheres (Figure 5D). The overexpression efficacy of surviving was proven by western blotting assay (Supplementary Figure S2A). In brief, all these results demonstrated that POS targets survivin to weaken CSCs stemness in GBM cells.

### Posaconazole weakens cancer stem-like cells stemness via the Wnt/β-catenin signaling pathway in glioblastoma cells

**FIGURE 6:**
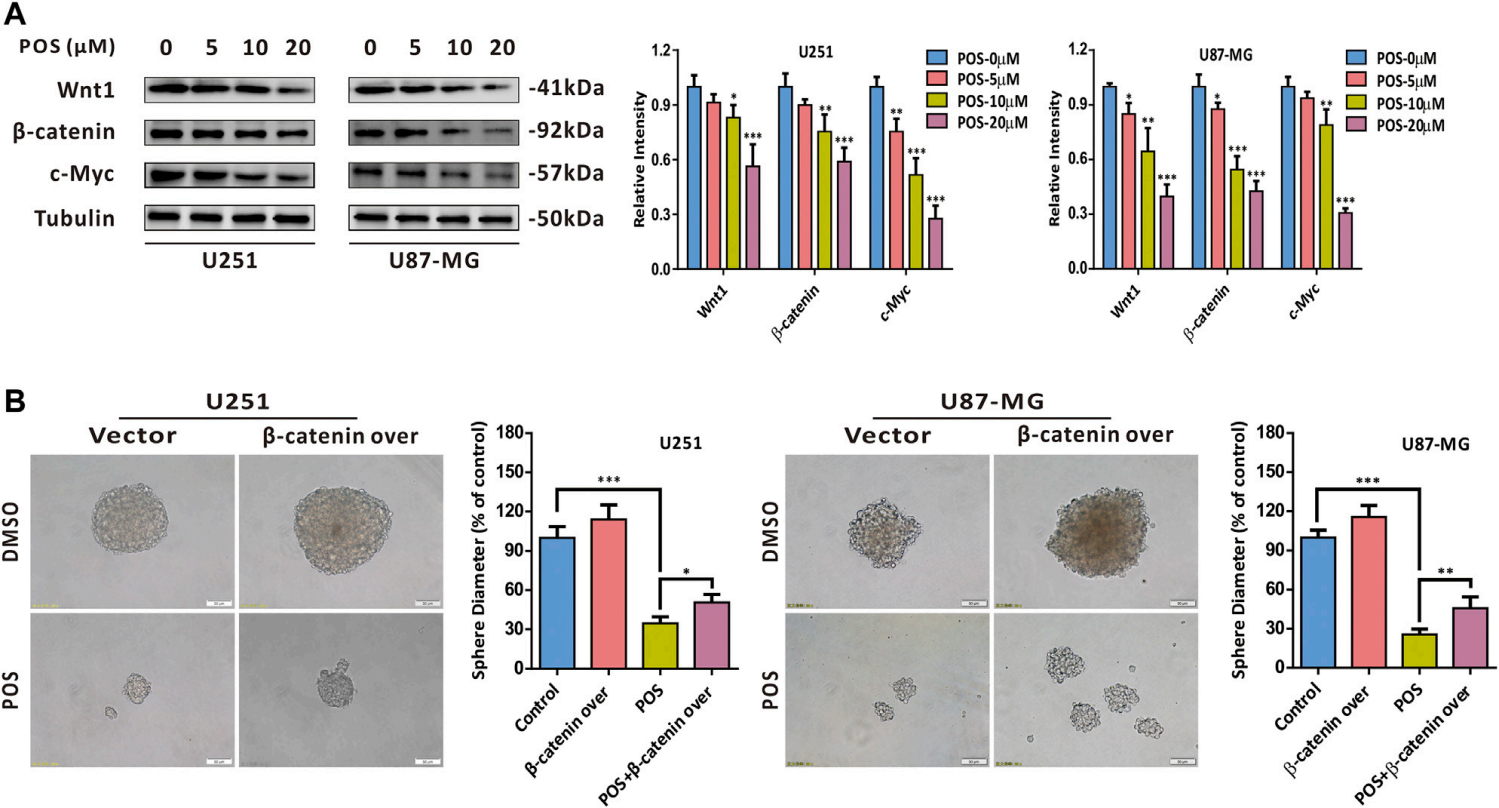
POS attenuates CSCs stemness *via* the Wnt/β-catenin signaling axis in GBM cells. **(A)** Expression levels of Wnt1, β-catenin and c-Myc were detected by western blotting after the cells were treated as indicated for 24 h. **(B)** Self-renewal capacity was detected by a sphere formation assay after the β-catenin-overexpressed or vector control cells were treated with POS or DMSO for 2 weeks. Scale bar, 50 μM.

To further screen out the potential signaling pathway by which POS inhibits CSCs stemness, we performed a gene set difference analysis (GSVA) comparing the high and low survivin expression datasets. As shown in Supplementary Figure S3, the GSVA showed that survivin was significantly correlated with the Wnt/β-Catenin, Notch and Hedgehog signaling pathways, which were previously reported to be involved in the stemness of cancer cell (Amakye et al., 2013; Liu et al., 2018). In addition, survivin was identified as a downstream protein of the Wnt/β-catenin/TCF/LEF signaling pathway by KEGG pathway analysis (Supplementary Figure S4). Therefore, we chose the Wnt/β-catenin signaling pathway as the candidate signaling pathway for POS in GBM cells. The western blot analysis indicated that POS dose-dependently decreased the expression levels of Wnt1, β-catenin and c-Myc (Figure 6A). Moreover, β-catenin overexpression partially counteracted the inhibitory effect of POS on the capacity to form spheres (Figure 6B). The western blotting analysis verified the efficiency of β-catenin overexpression (Supplementary Figure S2B). Taken together, these data demonstrated that POS attenuates CSCs stemness through inhibiting the Wnt/β-catenin signaling pathway in GBM cells.

### Posaconazole-induced autophagy intersects with the Wnt/β-catenin signaling pathway

**FIGURE 7:**
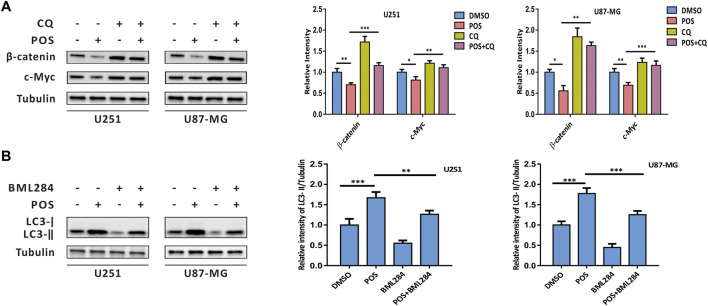
POS-induced autophagy intersects with the Wnt/β-catenin signaling pathway. **(A)** Expression levels of β-catenin and c-Myc were detected by western blotting after the cells were treated with or without POS in the presence or absence of CQ. **(B)** Expression levels of LC3 were measured by western blotting after the cells were treated with or without POS in the presence or absence of BML284.

To further explore the relationship of POS-induced autophagy with the Wnt/β-catenin signaling pathway, firstly, POS-induced autophagy was blocked by the autophagy inhibitor CQ to investigate the effect of autophagy on the Wnt/β-catenin signaling pathway. Western blot results showed that the down-regulation effect of POS on β-catenin and c-Myc expression was remarkably rescued by combination with CQ (Figure 7A). Conversely, when POS-inhibited Wnt/β-catenin pathway was activated by an activator of Wnt signaling BML284, western blot assay indicated that LC3-II expression was significantly decreased by POS plus BML284 compared to POS alone as evidence that POS-induced autophagy was inhibited by the activation of the Wnt signaling pathway (Figure 7B). In short, POS-induced autophagy and the Wnt/β-catenin signaling pathway are negative regulators for each other.

### Posaconazole reduces glioblastoma growth *in vivo*

**FIGURE 8:**
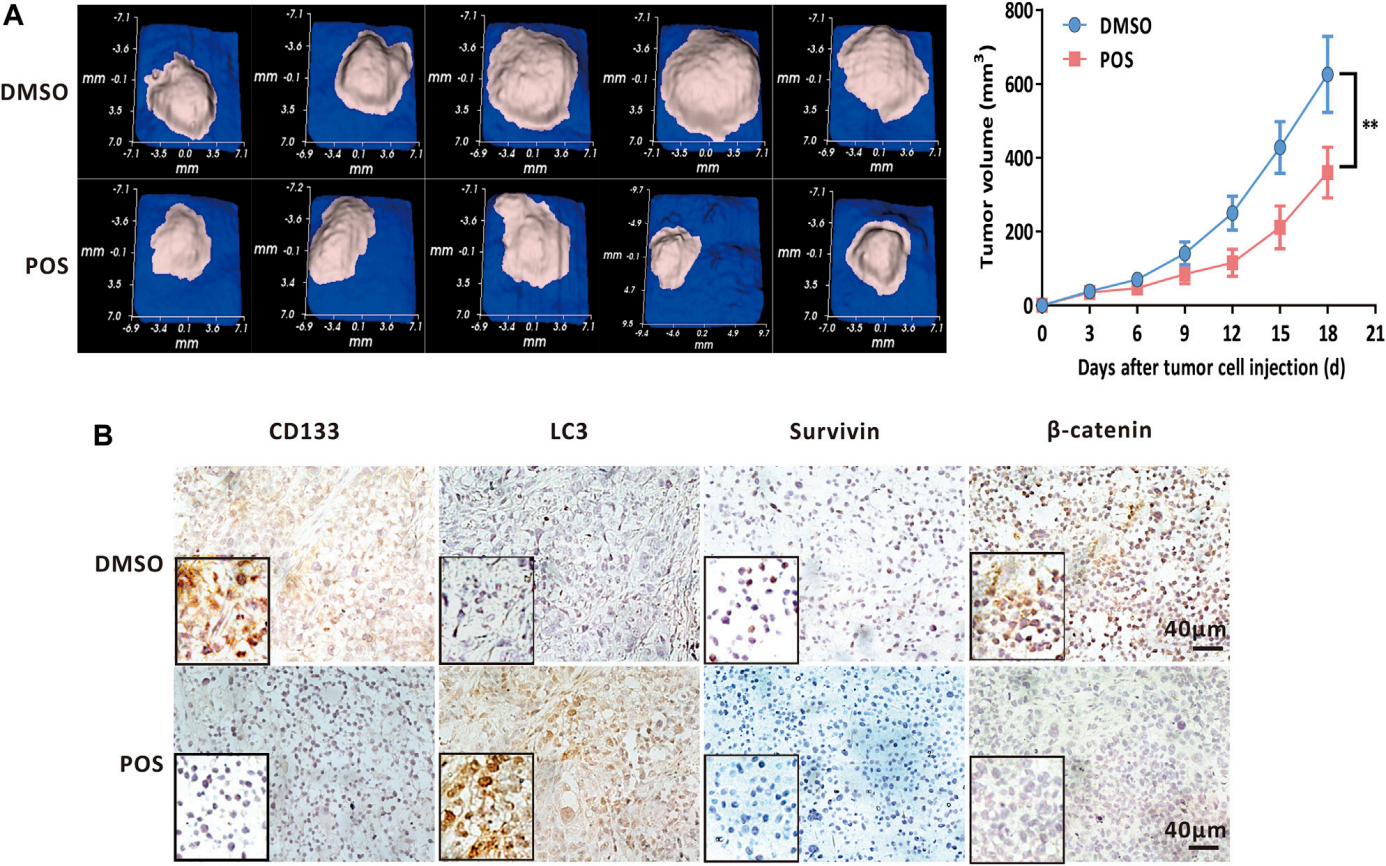
POS decreases GBM tumor growth *in vivo.* **(A)** U87-MG cells were subcutaneously implanted into immunodeficient nude mice. The mice were randomly divided into 2 groups and administered DMSO or POS at intervals of 3 days for a total of 6 administrations. The tumor volumes were detected every 3 days. Left, tumor images were photographed on the 18th day. Right, tumor volumes was measured at different time points (*n* = 5). **(B)** Expression of LC3, CD133, survivin and β-catenin in U87-MG xenografts that were collected from DMSO or POS-treated mice was measured by immunohistochemical analysis. Scale bar: 40 μM.

In light of the inhibitory effect of POS on GBM cells and CSCs stemness, we investigated whether POS could exert antitumor effects *in vivo*. To this end, we subcutaneously implanted U87-MG cells into immunodeficient nude mice. The mice were administered DMSO or POS at intervals of 3 days for a total of 6 times, and then the tumor volumes were detected every 3 days. As indicated in Figure 8A, the tumors of the POS group were significantly reduced compared with those of the DMSO control group. In addition, the immunohistochemistry analysis demonstrated that POS could enhance the expression of LC3 and decrease the expression of CD133, survivin and β-catenin, which was consistent with the *in vitro* results (Figure 8B). Collectively, these results clearly revealed that POS possesses antitumor activity against GBM *in vivo*.

## Discussion

The mainstay therapy for GBM includes surgery, radiation and adjuvant chemotherapy. GBM patients benefit from conventional treatment with prolonged survival time and improved quality of life. However, complete healing is rarely achieved. Treatment failure is highly relevant to CSCs. CSCs are a small subpopulation of cancer cells that have extraordinary self-renewal and differentiation capacities and are responsible for initiating and maintaining the tumor, which is why CSCs are also known as tumor-initiating cells or tumor-propagating cells. The hierarchical model has been proposed to explain tumor origin and heterogeneity, and it is based on the concept that CSCs induce tumor origin and lead to heterogeneity by generating differentiated and quiescent cancer cells. Therefore, CSCs are located at the top of the heterogeneity hierarchy. In general, CSCs survival or not appears critical for the chemotherapeutic efficacy of cancer treatment regimens. In this study, the results of the sphere formation assays, western blot analysis and flow cytometry analysis, which were employed to detect the self-renewal capacity, the expression levels of classical CSCs marker proteins (for example, CD133, SOX2, Nanog and Oct4) and the subpopulation of CD133^+^/CD44^+^ cells, showed that POS could significantly weaken CSCs stemness in GBM cells as evidence that POS is a potential CSCs-targeting antitumor drug for GBM treatment.

Autophagy is one of the crucial processes, that is, tightly involved in CSCs survival. Autophagy is a self-digestion process in which the cytoplasm, including some proteins, damaged organelles and lipids, is sequestered into autophagosomes for further degradation and recycling. Under basic conditions, autophagy is vital for maintaining cell homeostasis. Depending on the specific tumor context, such as the tumor type/stage, the local tumor microenvironment, and the specific cancer therapy treatment, autophagy possesses the opposite functions: tumor-suppression and tumor-promotion. Similarly, the opposite roles of autophagy were explained as being a double-edged sword in CSCs. On one hand, autophagy is essential for the maintenance and survival of CSCs. For example, autophagy is required for the pluripotency of CSCs (Sharif et al., 2017), and autophagy deficiency or inhibition results in the suppression of stem-like features and CSCs phenotypes (Zhang et al., 2016; Zhang et al., 2017). The combination of autophagy inhibitors could improve the efficacy of chemotherapeutics (Bellodiet al., 2009; Pagotto et al., 2017). On the other hand, autophagic induction could promote autophagic cell death or apoptosis of CSCs (Kumar et al., 2013). In this study, multiple detection methods for autophagy, including western blot assays to measure autophagy marker proteins, TEM to visually observe the ultrastructural morphology of autophagosomes and AO staining to monitor the formation of AVOs, indicated that POS could induce autophagy in GBM cells. Intriguingly, the inhibitory effect of POS on CSCs features was partially counteracted when autophagy was blocked regardless of whether Atg5 shRNA or an autophagy inhibitor was used. Thus, POS-induced autophagy performs suppressive function in CSCs stemness.

As a member of the inhibitor of apoptosis protein (IAP) family, survivin (also known as BRIC5) is highly expressed in cancer cells and CSCs (Altieri, 2008) but undetectable in normal cells. Survivin is an important oncogene and is widely involved in cell division, apoptosis, the cellular stress response, cell invasion and metastasis, which makes it a hotspot protein in cancer research (Altieri, 2013). Recent research revealed that survivin also plays a vital role in CSCs. For example, iron-saturated bovine lactoferrin nanocarriers/nanocapsules killed colon cancer stem cells by targeting survivin (Kanwar et al., 2015). Survivin is overexpressed in the cancer stem cell pool of doxorubicin-resistant breast cancer cells. Interestingly, survivin siRNA could inhibit stemness, eliminate cancer stem cells by apoptosis and finally reverse chemoresistance (Wang et al., 2015). In this study, we screened survivin as a target protein for POS in GBM cells based on an intersection of WGCNA analysis for RNA-sequencing gene expression profiles downloaded from TCGA with autophagy-related genes provided by the human autophagy database HADb. Moreover, western blot assays validated that POS could downregulate the protein expression levels of survivin in a dose-dependent manner in GBM cells. More importantly, as shown in the sphere formation assays, overexpression of survivin significantly offset the inhibitory effect of POS on CSCs self-renewal ability.

Accumulating evidence indicates that CSCs are regulated by key signaling pathways in GBM, such as Wnt, Hedgehog and Notch (Clevers and Nusse, 2006; Fan et al., 2010; Amakye et al., 2013; Liu et al., 2018). For example, the stemness and chemoresistance of circulating tumor cells (CTCs) were induced by activating Wnt in GBM (Liu et al., 2018). In this study, gene set difference analysis (GSVA) revealed that survivin was significantly associated with the Myc, Wnt, Notch and Hedgehog signaling pathways, which is consistent with previous research (Supplementary Figure S3). Furthermore, survivin was identified as the downstream protein of the Wnt/β-catenin/TCF/LEF signaling pathway by KEGG pathway analysis (Supplementary Figure S4). The expression levels of key proteins in the Wnt/β-catenin signaling pathway, including Wnt1, β-catenin and c-Myc, were decreased by POS, as shown by western blot assays. Furthermore, sphere formation assays verified that the self-renewal capacity was partially restored in POS-treated GBM cells when β-catenin was overexpressed.

The crosstalk between autophagy and the Wnt/β-catenin pathway has been verified in a large number of cellular processes (Wu, et al., 2013; Lorzadeh et al., 2021). β-catenin is found to contain an LC3 interacting motif. When autophagy is activated, the Wnt signaling pathway is repressed via LC3 binding with β-catenin and subsequent autophagic degradation of β-catenin (Petherick et al., 2013). Conversely, the inhibition of the Wnt/β-catenin pathway can induce autophagy machinery (Nàger et al., 2018). In this sense, autophagy and the Wnt/β-catenin pathway negatively regulate for each other. Western blot assay (Figure 7) in this study further confirmed the regulatory feedback model between POS-induced autophagy and the Wnt/β-catenin signaling pathway.

Finally, certain limitations in this study must be noted. This study demonstrated that POS-induced autophagy participates in the suppressive effect of POS on CSCs stemness in GBM cells. However, the molecular mechanisms by which POS-induced autophagy crosstalks with CSCs remain unclear. In addition, this work showed that POS could weaken CSCs stemness in GBM cells. Given the role of CSCs in initiating and maintaining the tumor, it is necessary to explore the chemotherapy resistance of POS for GBM treatment. Therefore, considerable effort should be made to solve these questions in future research work to reveal a complete story.

## Conclusion

**FIGURE 9:**
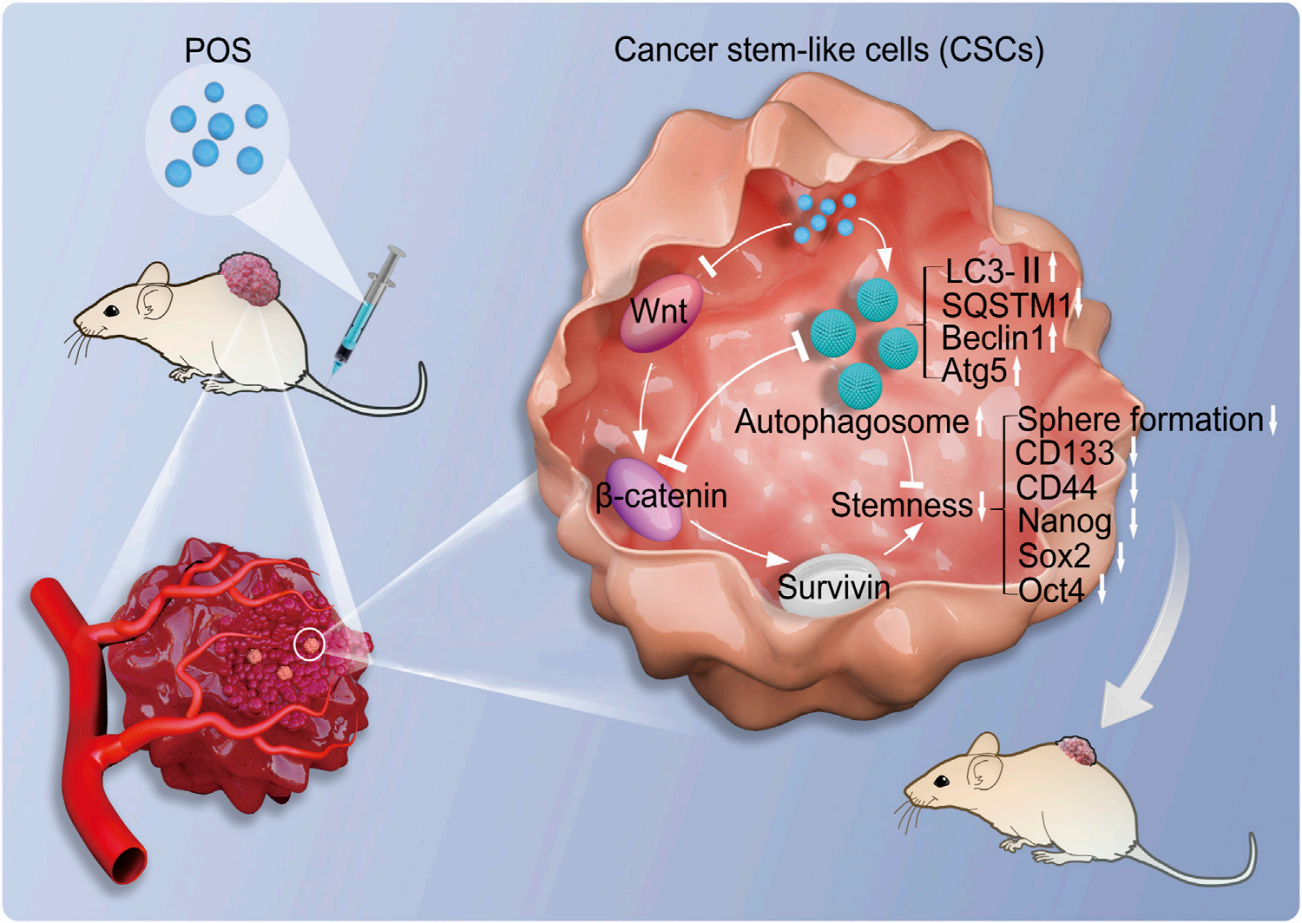
Molecular mechanisms of POS weakens CSCs stemness in GBM. POS inhibits CSCs stemness by autophagy induction and suppressing the Wnt/β-catenin/survivin signaling pathway.

In summary, this study investigated the antitumor molecular mechanisms of POS. As indicated in Figure 9, this work demonstrated that POS remarkably inhibits CSCs stemness and induces autophagy in GBM cells. Importantly, the suppressive effect of POS on CSCs stemness was partially relieved when autophagy was blocked. By combining bioinformatic analysis with experimental validation, we verified that POS weakened CSCs stemness by downregulating survivin and suppressing the Wnt/β-catenin signaling pathway. Moreover, POS-induced autophagy and the Wnt/β-catenin signaling pathway negatively regulates for each other. The animal study further confirmed the antitumor activity of POS *in vivo*. Therefore, this work offers an experimental foundation for exploiting POS as a CSCs-targeting antitumor drug for GBM treatment.

## Data Availability

The original contributions presented in the study are included in the article/Supplementary Material, further inquiries can be directed to the corresponding author.

## Abbreviations

POS, posaconazole; GBM, glioblastoma; CSCs, cancer stem-like cells; CQ, chloroquine; WGCNA, weighted gene coexpression network analysis; GSVA, gene set difference analysis; KEGG, Kyoto Encyclopedia of Genes and Genomes; AO, acridine orange; ANOVA, one-way analysis of variance; TEM, transmission electron microscopy; PVDF, polyvinylidene difluoride; DAPI, 4′,6-diamidino-2-phenylindole.

## References

[B1] Agnihotri S. Mansouri S. Burrell K. Li M. Mamatjan Y. Liu J. (2019). Ketoconazole and posaconazole selectively target HK2-expressing glioblastoma cells. Clin. Cancer Res. 25, 844–855. 10.1158/1078-0432.CCR-18-1854 30322879 PMC8103287

[B2] Altieri D. C. (2008). Survivin, cancer networks and pathway-directed drug discovery. Nat. Rev. Cancer 8, 61–70. 10.1038/nrc2293 18075512

[B3] Altieri D. C. (2013). Targeting survivin in cancer. Cancer Lett. 332, 225–228. 10.1016/j.canlet.2012.03.005 22410464 PMC3695618

[B4] Amakye D. Jagani Z. Dorsch M. (2013). Unraveling the therapeutic potential of the Hedgehog pathway in cancer. Nat. Med. 19, 1410–1422. 10.1038/nm.3389 24202394

[B5] Anton K. Baehring J. M. Mayer T. (2012). Glioblastoma multiforme: overview of current treatment and future perspectives. Hematol. Oncol. Clin. North Am. 26, 825–853. 10.1016/j.hoc.2012.04.006 22794286

[B6] Bellodi C. Lidonnici M. R. Hamilton A. Helgason G. V. Soliera A. R. Ronchetti M. (2009). Targeting autophagy potentiates tyrosine kinase inhibitor-induced cell death in Philadelphia chromosome-positive cells, including primary CML stem cells. J. Clin. Invest.. 119, 1109–1123. 10.1172/JCI35660 19363292 PMC2673867

[B7] Brown D. V. Daniel P. M. D'Abaco G. M. Gogos A. Ng W. Morokoff A. P. (2015). Coexpression analysis of CD133 and CD44 identifies proneural and mesenchymal subtypes of glioblastoma multiforme. Oncotarget 6, 6267–6280. 10.18632/oncotarget.3365 25749043 PMC4467436

[B8] Brown D. V. Filiz G. Daniel P. M. Hollande F. Dworkin S. Amiridis S. (2017). Expression of CD133 and CD44 in glioblastoma stem cells correlates with cell proliferation, phenotype stability and intra-tumor heterogeneity. PLoS One 12, e0172791. 10.1371/journal.pone.0172791 28241049 PMC5328356

[B9] Chen J. Li Y. Yu T. S. McKay R. M. Burns D. K. Kernie S. G. (2012). A restricted cell population propagates glioblastoma growth after chemotherapy. Nature 488, 522–526. 10.1038/nature11287 22854781 PMC3427400

[B10] Clevers H. Nusse R. (2006). Wnt/beta-catenin signaling in development and disease. Cell 127, 469–480. 10.1016/j.cell.2006.10.018 17081971

[B11] Eyler C. E. Wu Q. Yan K. MacSwords J. M. Chandler-Militello D. Misuraca K. L. (2011). Glioma stem cell proliferation and tumor growth are promoted by nitric oxide synthase-2. Cell 146, 53–66. 10.1016/j.cell.2011.06.006 21729780 PMC3144745

[B12] Fan X. Khaki L. Zhu T. S. Soules M. E. Talsma C. E. Gul N. (2010). NOTCH pathway blockade depletes CD133-positive glioblastoma cells and inhibits growth of tumor neurospheres and xenografts. Stem Cells 28, 5–16. 10.1002/stem.254 19904829 PMC2878196

[B13] Flavahan W. A. Wu Q. Hitomi M. Rahim N. Kim Y. Sloan A. E. (2013). Brain tumor initiating cells adapt to restricted nutrition through preferential glucose uptake. Nat. Neurosci. 16, 1373–1382. 10.1038/nn.3510 23995067 PMC3930177

[B14] Kanwar J. R. Mahidhara G. Roy K. Sasidharan S. Krishnakumar S. Prasad N. (2015). Fe-bLf nanoformulation targets survivin to kill colon cancer stem cells and maintains absorption of iron, calcium and zinc. Nanomedicine 10, 35–55. 10.2217/nnm.14.132 25017148

[B15] Kumar D. Shankar S. Srivastava R. K. (2013). Rottlerin-induced autophagy leads to the apoptosis in breast cancer stem cells: molecular mechanisms. Mol. Cancer 12, 171. 10.1186/1476-4598-12-171 24359639 PMC3914415

[B16] Liu T. Xu H. Huang M. Ma W. Saxena D. Lustig R. A. (2018). Circulating glioma cells exhibit stem cell-like properties. Cancer Res. 78, 6632–6642. 10.1158/0008-5472.CAN-18-0650 30322863 PMC6497085

[B17] Lorzadeh S. Kohan L. Ghavami S. Azarpira N. (2021). Autophagy and the Wnt signaling pathway: a focus on wnt/β-catenin signaling. Biochim. Biophys. Acta. Mol. Cell Res. 1868, 118926. 10.1016/j.bbamcr.2020.118926 33316295

[B18] Lyne S. B. Yamini B. (2021). An alternative pipeline for glioblastoma therapeutics: a systematic review of drug repurposing in glioblastoma. Cancers (Basel) 13, 1953. 10.3390/cancers13081953 33919596 PMC8073966

[B19] Malta T. M. Sokolov A. Gentles A. J. Burzykowski T. Poisson L. Weinstein J. N. (2018). Machine learning identifies stemness features associated with oncogenic dedifferentiation. Cell 173, 338. 10.1016/j.cell.2018.03.034 l 29625051 PMC5902191

[B20] Marino G. Niso-Santano M. Baehrecke E. H. Kroemer G. (2014). Self-consumption: the interplay of autophagy and apoptosis. Nat. Rev. Mol. Cell Biol. 15, 81–94. 10.1038/nrm3735 24401948 PMC3970201

[B21] Nàger M. Sallán M. C. Visa A. Pushparaj C. Santacana M. Macià A. (2018). Inhibition of WNT-CTNNB1 signaling upregulates SQSTM1 and sensitizes glioblastoma cells to autophagy blockers. Autophagy 14, 619–636. 10.1080/15548627.2017.1423439 29313411 PMC5959333

[B22] Pagotto A. Pilotto G. Mazzoldi E. L. Nicoletto M. O. Frezzini S. Pasto A. (2017). Autophagy inhibition reduces chemoresistance and tumorigenic potential of human ovarian cancer stem cells. Cell Death Dis. 8, e2943. 10.1038/cddis.2017.327 28726781 PMC5550872

[B23] Petherick K. J. Williams A. C. Lane J. D. Ordóñez-Morán P. Huelsken J. Collard T. J. (2013). Autolysosomal β-catenin degradation regulates Wnt-autophagy-p62 crosstalk. EMBO J. 32, 1903–1916. 10.1038/emboj.2013.123 23736261 PMC3981178

[B24] Poser S. W. Otto O. Arps-Forker C. Ge Y. Herbig M. Andree C. (2019). Controlling distinct signaling states in cultured cancer cells provides a new platform for drug discovery. FASEB J. 33, 9235–9249. 10.1096/fj.201802603RR 31145643

[B25] Reya T. Morrison S. J. Clarke M. F. Weissman I. L. (2001). Stem cells, cancer, and cancer stem cells. Nature 414, 105–111. 10.1038/35102167 11689955

[B26] Schein R. Homans J. Larsen R. A. Neely M. (2011). Posaconazole for chronic refractory coccidioidal meningitis. Clin. Infect. Dis. 53, 1252–1254. 10.1093/cid/cir734 21987729

[B27] Sharif T. Martell E. Dai C. Kennedy B. E. Murphy P. Clements D. R. (2017). Autophagic homeostasis is required for the pluripotency of cancer stem cells. Autophagy 13, 264–284. 10.1080/15548627.2016.1260808 27929731 PMC5324853

[B28] Stupp R. Hegi M. E. Mason W. P. van den Bent M. J. Taphoorn M. J. Janzer R. C. (2009). Effects of radiotherapy with concomitant and adjuvant temozolomide versus radiotherapy alone on survival in glioblastoma in a randomised phase III study: 5-year analysis of the EORTC-NCIC trial. Lancet. Oncol. 10, 459–466. 10.1016/S1470-2045(09)70025-7 19269895

[B29] Stupp R. Mason W. P. van den Bent M. J. Weller M. Fisher B. Taphoorn M. J. (2005). Radiotherapy plus concomitant and adjuvant temozolomide for glioblastoma. N. Engl. J. Med. 352, 987–996. 10.1056/NEJMoa043330 15758009

[B30] Stupp R. Taillibert S. Kanner A. Read W. Steinberg D. Lhermitte B. (2017). Effect of tumor-treating fields plus maintenance temozolomide vs maintenance temozolomide alone on survival in patients with glioblastoma: a randomized clinical trial. JAMA 318, 2306–2316. 10.1001/jama.2017.18718 29260225 PMC5820703

[B31] University Health Network (2018). Azoles targeting recurrent high grade gliomas. Available at: https://clinicaltrials.gov/ct2/show/NCT03763396 (Accessed February 1, 2021).

[B32] Wang T. Gantier M. P. Xiang D. Bean A. G. Bruce M. Zhou S. F. (2015). EpCAM aptamer-mediated survivin silencing sensitized cancer stem cells to doxorubicin in a breast cancer model. Theranostics 5, 1456–1472. 10.7150/thno.11692 26681989 PMC4672025

[B33] Wang Y. Xu H. Liu T. Huang M. Butter P. P. Li C. (2018). Temporal DNA-PK activation drives genomic instability and therapy resistance in glioma stem cells. JCI Insight 3, e98096. 10.1172/jci.insight.98096 PMC5821187 29415883

[B34] Wu X. Won H. Rubinsztein D. C. (2013). Autophagy and mammalian development. Biochem. Soc. Trans. 41, 1489–1494. 10.1042/BST20130185 24256242

[B35] Zhang D. Zhao Q. Sun H. Yin L. Wu J. Xu J. (2016). Defective autophagy leads to the suppression of stem-like features of CD271^+^ osteosarcoma cells. J. Biomed. Sci. 23, 82. 10.1186/s12929-016-0297-5 27863492 PMC5116184

[B36] Zhang L. Xu L. Zhang F. Vlashi E. (2017). Doxycycline inhibits the cancer stem cell phenotype and epithelial-to-mesenchymal transition in breast cancer. Cell Cycle 16, 737–745. 10.1080/15384101.2016.1241929 27753527 PMC5405729

