# Supplementary figures and images for: Posaconazole inhibits the stemness of cancer stem-like cells by inducing autophagy and suppressing the Wnt/β-catenin/survivin signaling pathway in glioblastoma
Source: Front Pharmacol. 2022 Aug 11;13:905082. doi: 10.3389/fphar.2022.905082 (PMC9403519; doi:10.3389/fphar.2022.905082)

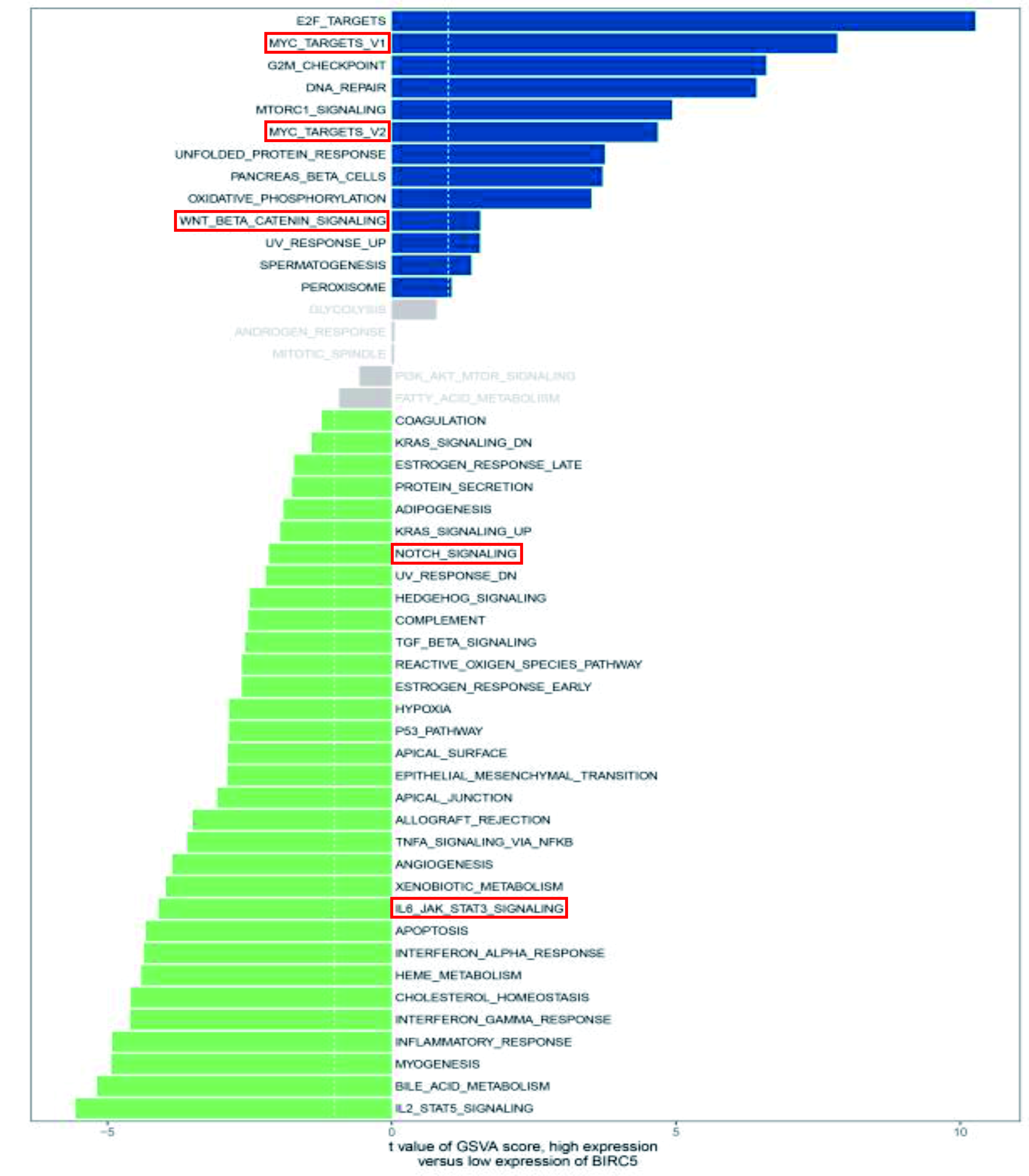

Supplement: Supplementary file 1 [file Image3.JPEG]

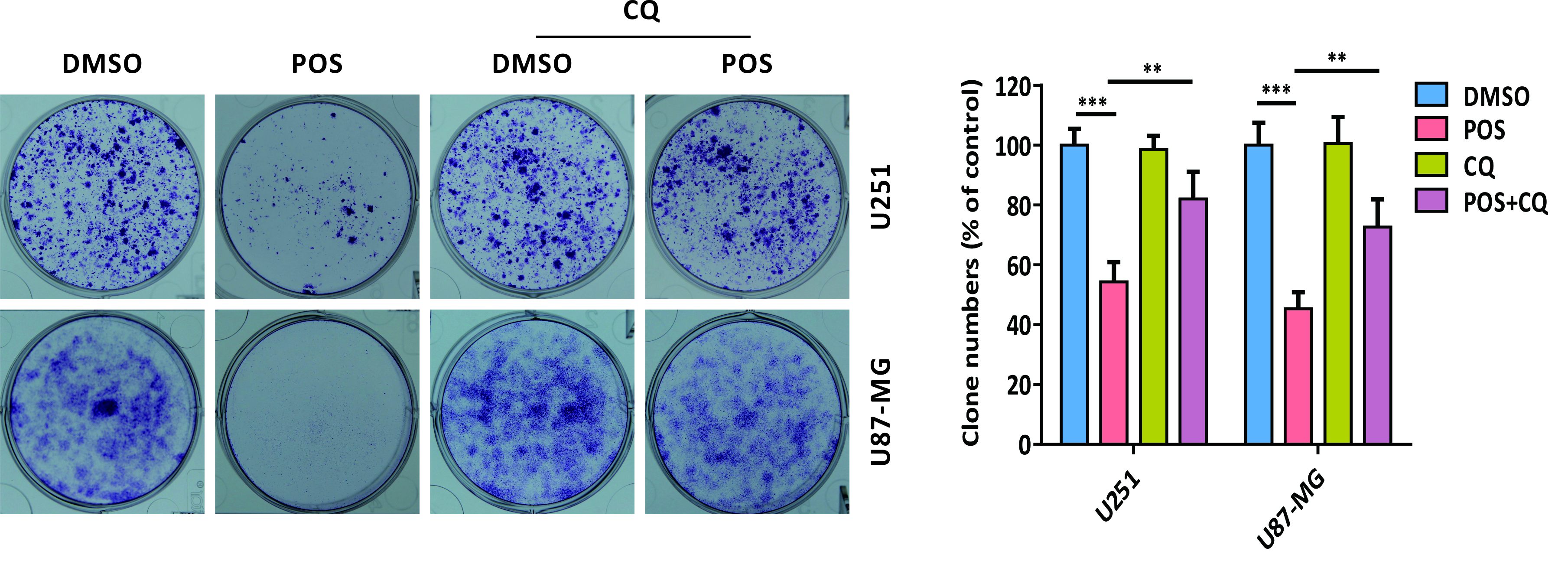

Supplement: Supplementary file 2 [file Image1.JPEG]

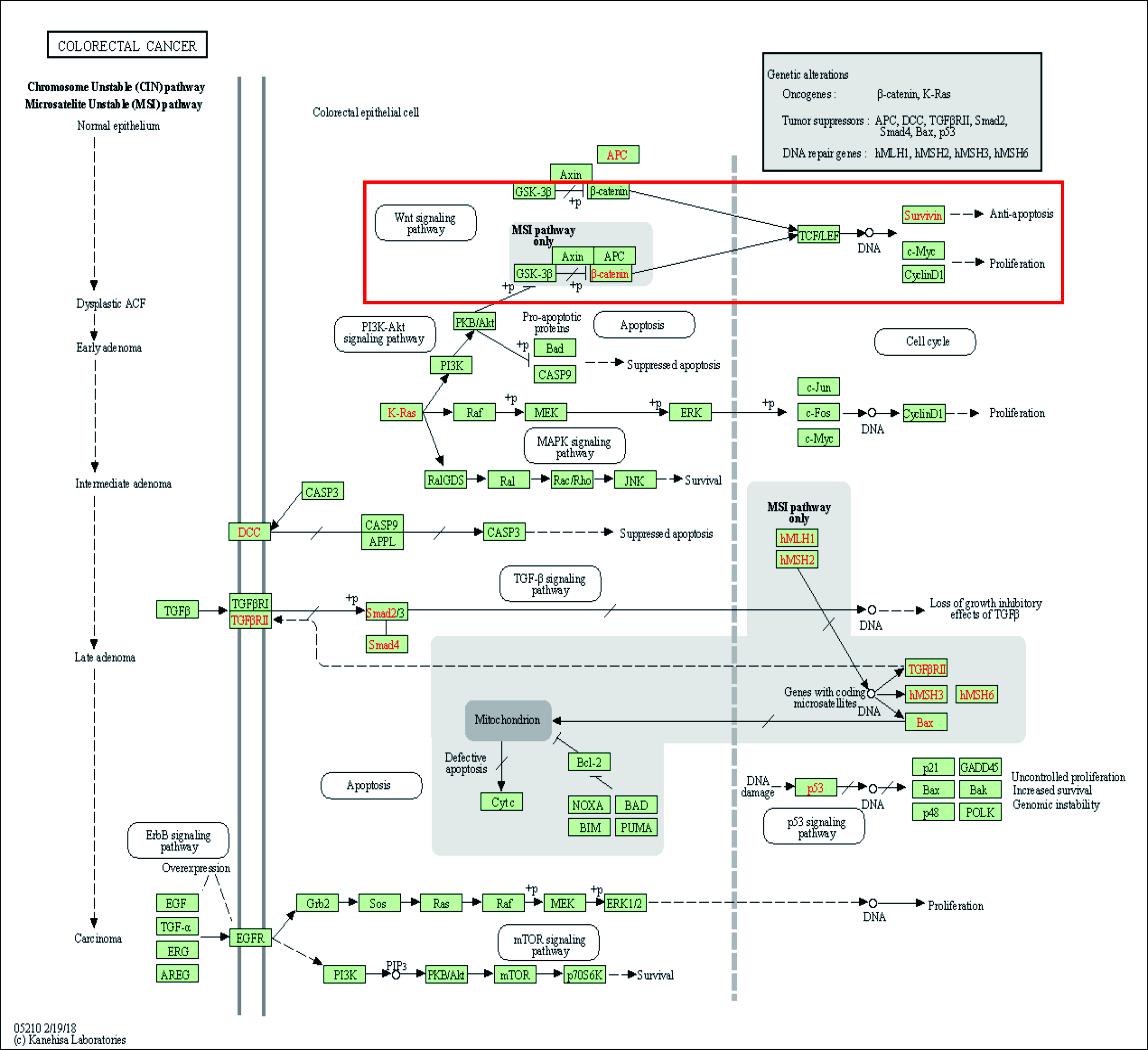

Supplement: Supplementary file 3 [file Image4.JPEG]

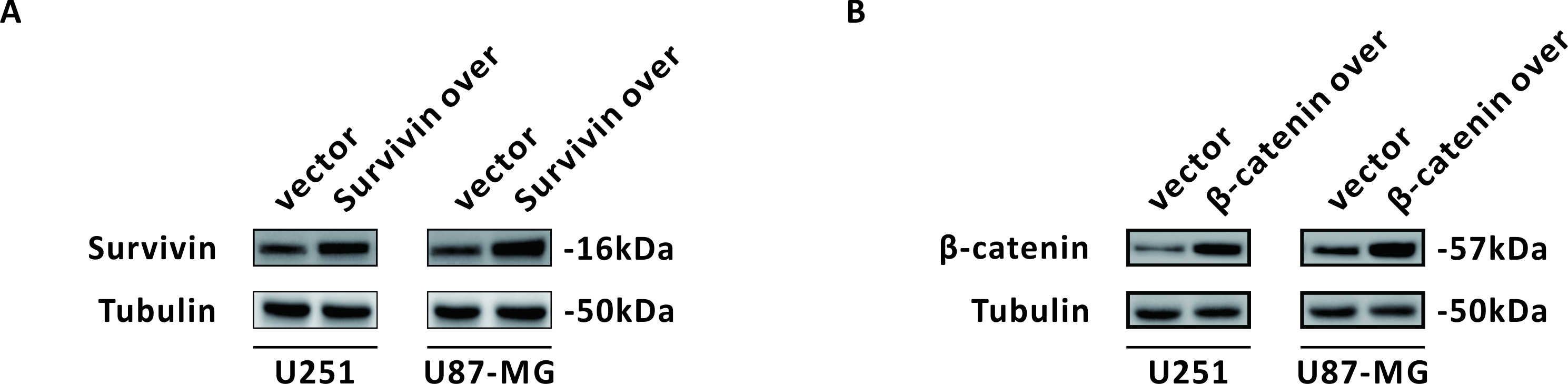

Supplement: Supplementary file 4 [file Image2.JPEG]
